# Primary B Cell Lymphoma of the CNS Mimicking Anti-LGI1 Limbic Encephalitis

**DOI:** 10.3389/fneur.2018.00658

**Published:** 2018-08-10

**Authors:** Christian Thomas, Christoph Lehrich, Catharina C. Gross, Heinz Wiendl, Sven G. Meuth, Nico Melzer

**Affiliations:** ^1^Clinic of Neurology with Institute of Translational Neurology, University of Münster, Münster, Germany; ^2^Institute of Neuropathology, University of Münster, Münster, Germany

**Keywords:** PCNSL, LE, MRI, antibodies, diagnostic criteria

## Abstract

Limbic encephalitis is a potentially paraneoplastic type of encephalitis mainly involving the limbic system. Recently, diagnostic criteria comprising clinical presentation as well as imaging, laboratory and electrophysiological findings have been established. Here, we show that incipient primary central nervous system lymphoma can closely resemble limbic encephalitis including positive testing for anti-LGI1 antibodies illustrating the need for thorough interpretation of initial laboratory and radiologic findings and tight follow-up examinations.

## Introduction

Limbic encephalitis (LE) is a potentially paraneoplastic type of encephalitis mainly involving the limbic system. According to recently established criteria (1), autoimmune LE can be diagnosed in patients with (i) subacute onset (rapid progression of < 3 months) of memory deficits, seizures, or psychiatric symptoms suggesting involvement of the limbic system that exhibit (ii) bilateral abnormalities (signal and volume increase) on T2-weighted fluid-attenuated inversion recovery (FLAIR) magnet resonance imaging (MRI) restricted to the medial temporal lobes together with (iii) either cerebrospinal fluid (CSF) pleocytosis or electroencephalography (EEG) showing epileptic and/or slow-wave activity involving the temporal lobes. Moreover, (iv) alternative causes need to be reasonably excluded. If one of the first three criteria is not met, a diagnosis of definite LE can be made only with the detection of antibodies against cell-surface, synaptic, or onconeural proteins. Here, we show that incipient primary central nervous system lymphoma (PCNSL) can closely resemble LE illustrating the need for tight clinical and paraclinical follow-up.

## Patient case

**Figure 1:**
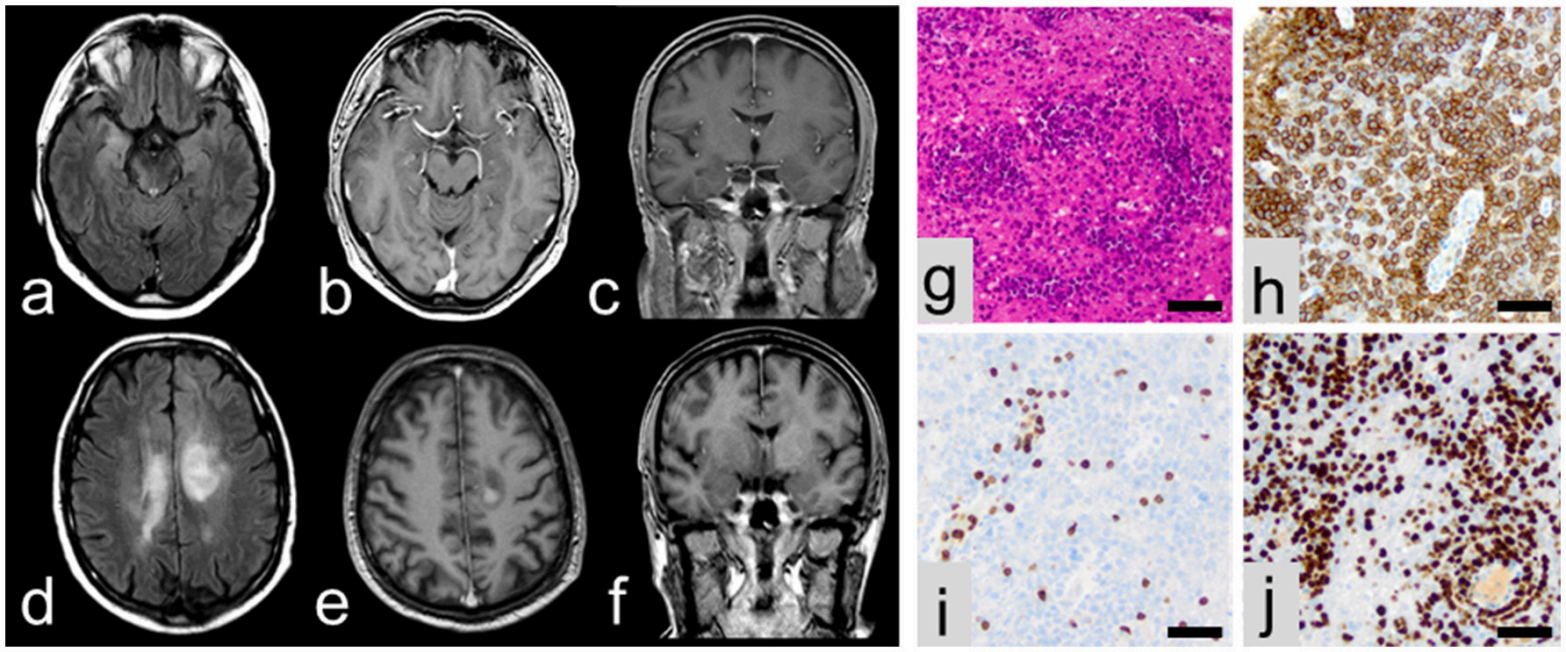
MRI at initial presentation showing volume and signal increase of the right medial temporal lobe with focal extension to neocortical areas on T2/FLAIR images **(a)** without contrast enhancement **(b,c)**. In contrast, follow-up MRI showing multiple lesions in axial T2/FLAIR **(d)** with contrast enhancement **(e,f)**. On histopathological examination, a malignant lymphoid tumor **(g)** with expression of CD20 **(h)** and some intermingled reactive CD3-positive lymphocytes **(i)** was encountered. Ki67/Mib1 proliferation index accounted for 92% **(j)**. All scale bars represent 50 μm.

A 61-year old left-handed caucasian male was admitted for a first unprovoked generalized tonic clonic epileptic seizure and recent memory disturbances (4 weeks prior to hospitalization). Past medical history included non-metastatic prostate carcinoma as well as progressive bilateral loss of vision which was diagnosed as chorioretinitis 1 month prior to admission. Dietary history was significant for unwanted weight loss of 10 kg over the last 6 weeks. Neurological examination was normal. Cerebral MRI showed volume and signal increase within right medial temporal lobe with focal extension to neocortical areas on T2/FLAIR images without contrast enhancement (Figures 1a–c). Moreover, non-contrast-enhancing T2/FLAIR-hyperintense signals were detected in the posterior leg of the right internal capsule extending to the right cerebral crus (Figure 1a), the right lentiform nucleus and the left corona radiata. CSF analysis revealed slight lymphocytic pleocytosis (8/μl) and dysfunction of the blood-CSF barrier (albumin ratio 8.3 × 10^−3^) with normal protein levels (448 mg/l). Flow cytometry (2, 3) showed a normal intrathecal CD4/CD8 ratio with elevated fractions of activated CD4^+^ and CD8^+^ T-cells but normal fractions of CD19^+^ B cells and no CD138^+^ plasma cells. There was no intrathecal immunoglobuline (Ig) synthesis and no CSF-specific oligoclonal bands (type 4 pattern). Testing for neuronal antibodies revealed anti-LGI1 IgG antibodies (4) in serum (titer 1:10), but not in CSF. EEG showed intermittent polymorph slowing in the delta band (1–2/s) together with intermittent sharp waves and sharp-slow-waves over the right anterior temporal lobe. Neuropsychological assessment demonstrated severe verbal but not figural memory deficits consistent with right temporal findings on MRI and EEG together with impaired executive abilities and attention span. There was no evidence of systemic or cerebral infections, and a CT examination of the chest and abdomen as well as bone scintigraphy did not show any signs of metastasis of the prostate cancer or any other malignancy. Based on these findings, the patient was diagnosed with anti-LGI1 LE and treated with tryptophan-immunoadsorption (6 cycles each with treatment of a plasma volume of 1.5–2.5 liters) followed by methylprednisolone pulse therapy (1 g/d over 5 days) as well as levetiracetam resulting in clinical improvement. Prostatectomy was performed 4 days after completion of the immunotherapy. Unexpectedly, the patient developed severe delirium and subsequent loss of consciousness during the postoperative course. CSF examination showed unchanged findings, but MRI revealed progression of the previously described non-contrast enhancing T2/FLAIR lesions with new contrast-enhancing lesions adjacent to the posteriorhorn of the right lateral ventricle and central of the left parietal lobe (Figures 1d–f). Biopsy from a contrast-enhancing left precentral lesion revealed a malignant lymphoid tumor of high cellularity composed of blastic cells with large pleomorphic nuclei (Figures 1g–j). Tumor cells showed both diffuse and angiocentric infiltration pattern. Neoplastic cells were positive for CD45 and CD20, while CD3 expression was restricted to few intermingled reactive lymphocytes. The Ki67/Mib1 proliferation index was high (92%). Based on these findings, diagnosis of diffuse large B-cell lymphoma was established. The patient was treated with a high-dose methotrexate-based chemotherapy followed by whole brain irradiation and succumbed to the disease 20 months after diagnosis.

## Discussion

In summary, this case illustrates that incipient PCNSL can resemble clinical and paraclinical features of LE including positive testing for neuronal antibodies. Red flags of this case include: unilateral medial temporal lobe involvement (on MRI, EEG and neuropsychological assessment) with concomitant (non-contrast enhancing) T2/FLAIR signal alterations outside the medial temporal lobe together with low titers of antibodies. Although diagnostic criteria for LE require lesion involvement confined to the medial temporal lobes (1), involvement of other structures such as basal ganglia has been reported in some cases of definite autoimmune encephalitis with LGI1 antibodies, but mostly in the context of faciobrachial dystonic seizures (FBDS) (5–7). However, involvement of corona radiata or crus cerebri has not been reported in association with LE. The detection of specific autoantibodies is crucial for establishing a definitive diagnosis of autoimmune encephalitis and identifies immunological subtypes of LE. Especially, antibodies against LGI1 and contactin associated protein 2 (CASPR2) have well-defined syndrome associations. While there have been no studies about the specificity of low serum LGI1 titers, a recent investigation in patients with CASPR2 antibodies suggests that only high CASPR2 serum antibody titers (i.e., ≥1:200) are of significant diagnostic value for anti-CASPR2 encephalitis (8). Therefore, further studies are needed to identify cut-off titers for LGI1 LE and the prevalence of LGI1 titers in PCNSL patients. In our case report, histopathological examination of a stereotactic biopsy lead to the diagnosis of PCNSL. In this context, prior administration of corticosteroids is the major diagnostic challenge in histopathological examination, because lymphoma cells are highly sensitivity to corticosteroid-induced apoptosis. Therefore administration of corticosteroids can mask the morphology and may even cause tumor vanishing (9). Taken together, thorough interpretation of initial laboratory and radiologic findings and tight follow-up examinations are warranted.

## Author contributions

CT acquisition of data, analysis and interpretation of data, writing of the manuscript. CL acquisition of data, writing of the manuscript. CG acquisition and interpretation of data, critical revision of manuscript for intellectual content. HW and SM critical revision of manuscript for intellectual content. NM study concept and design, critical revision of manuscript for intellectual content.

### Conflict of interest statement

The authors declare that the research was conducted in the absence of any commercial or financial relationships that could be construed as a potential conflict of interest.
